# Resource limitation determines realized thermal performance of consumers in trophodynamic models

**DOI:** 10.1111/ele.14086

**Published:** 2022-08-27

**Authors:** Anna C. Vinton, David A. Vasseur

**Affiliations:** ^1^ Department of Biology University of Oxford Oxford UK; ^2^ Department of Ecology and Evolutionary Biology Yale University New Haven Connecticut USA

**Keywords:** consumer‐resource model, metabolic meltdown, resource limitation, thermal performance

## Abstract

Recent work has demonstrated that changes in resource availability can alter a consumer's thermal performance curve (TPC). When resources decline, the optimal temperature and breadth of thermal performance also decline, leading to a greater risk of warming than predicted by static TPCs. We investigate the effect of temperature on coupled consumer‐resource dynamics, focusing on the potential for changes in the consumer TPC to alter extinction risk. Coupling consumer and resource dynamics generally reduces the potential for resource decline to exacerbate the effects of warming via changes to the TPC due to a reduction in top‐down control when consumers near the limits of their thermal performance curve. However, if resources are more sensitive to warming, consumer TPCs can be reshaped by declining resources, leading to increased extinction risk. Our work elucidates the role of top‐down and bottom‐up regulation in determining the extent to which changes in resource density alter consumer TPCs.

## Graphical abstract

We investigate the relationship between resource density and temperature (warming) on the persistence of a consumer population. Our work elucidates the importance of jointly considering temperature and resource limitation when assessing the thermal performance of species. We demonstrate how knowledge of the thermal performance of a resource population can be used to generate realized consumer thermal performance curves.

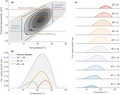

## INTRODUCTION

Given the quickening pace of environmental change marking the Anthropocene (Zalasiewicz et al., 2017), the investigation of how species will respond to temperature change has become increasingly important. This includes not only responses at the species level but at the community level (Urban et al., 2016) where inter‐specific interactions may change, be newly generated or disappear entirely. Much of the recent work on the impact of temperature warming and variability has relied upon the use of thermal performance curves (TPCs). TPCs describe the response of some fitness metric across a temperature gradient, but with temperature held constant during the assessment of performance (Angilletta, 2009; Bennett & Lenski, 1993; Huey & Slatkin, 1976; Kingsolver, 2009). These curves are commonly unimodal with the mode, or optimal value ($T_{\mathrm{opt}}$), skewed toward a warmer temperature. The lower and upper thermal limits of performance are defined as $CT_{\min}$ and ${\mathrm{CT}}_{\max}$ for physiological traits such as movement rate or response to stimuli, and as $T_{\min}$ and $T_{\max}$ for population demographic traits such as growth rate. In the latter case, the difference $T_{\max}-T_{\min}$ (often denoted ‘thermal breadth’) is consistent with Hutchinson's definition of the niche provided that resources are non‐limiting (Holt, 2009). Given the relative ease with which TPCs and thermal limits can be measured under both field and laboratory conditions, they have become a central tool for forecasting climate change impacts on ectotherms (Deutsch et al., 2008; Pearson & Dawson, 2003; Pinsky et al., 2019; Sinclair et al., 2016; Sunday et al., 2014; Vasseur et al., 2014).

Despite the prominence of TPCs in recent forecasting work, they are not well suited to this task. TPCs are generally measured under idealized conditions that are not common in nature (Schulte et al., 2011). Although numerous studies have sought to improve the link between TPCs and predictions of fitness in natural environments (Fey et al., 2019; Khelifa et al., 2019; Kremer et al., 2018; Sinclair et al., 2016), these have mainly focused on the effects of life‐history, behaviour and physiology. Recent work has shown that thermal performance curves can also respond to changes in the availability of resources such as food or light (Theus et al., 2022; Thomas et al., 2017) further challenging the practice of making a prediction based on ‘idealized’ TPCs. Building on the work of Brett et al. (1969) and Brett (1971), who showed that the individual growth of salmon was optimized at lower temperatures as the availability of food declined, Thomas et al. (2017) found that the optimal temperature for phytoplankton population growth occurred at lower temperatures under reduced nutrient availability and used a model to derive a mechanistic understanding of this shift. This implies that nutrient‐poor conditions may exacerbate the negative effects of warming by shifting the optimum (and upper threshold T_max_) towards cooler temperatures. This shift in the optimum temperature for growth could have additional detrimental consequences for populations if the mismatch between optimal and ambient temperatures leads to behavioural responses. Huey and Kingsolver (2019) suggested that a shifted optimal temperature could result in additional costs if individuals have to restrict their foraging and activity patterns to regulate their body temperature to match a cooler optimum. They coined the term ‘metabolic meltdown’ to describe the potential for warmer temperatures, a cooler thermal optimum, and thermally‐imposed restrictions on activity, to exacerbate the detrimental effects of warming when temperatures exceed preferred body temperatures.

Critical to the idea that a shift in the optimum temperature for performance could exacerbate the risk of warming (via a shift in the TPC or via a ‘metabolic meltdown’) is the assumption that food resources or nutrients will decline in concert with warming. There is growing evidence that warming is reducing the availability of food resources for herbivores, particularly when warming is coupled with changes in precipitation (Pan et al., 2017; Lister & Garcia, 2018; and see Huey & Kingsolver, 2019). Moreover, numerous studies support the prediction that warming is reducing the performance of many species of ectotherms, creating the possibility for additional resource limitations amongst their predators (Deutsch et al., 2008; Sherry, 2021). Previous work considering the coupled impact of warming and resource availability has assumed that resource densities are independent of consumers (Huey & Kingsolver, 2019; Thomas et al., 2017) and, therefore, that any effect of warming on consumers would be independent of the effect that they experience indirectly via changes in resource density. However, for many systems, it may be unreasonable to uncouple these responses. The pressure on resources that are generated by consumption generates a dynamic interplay that has been studied for over a century and is responsible for important phenomena such as predator–prey cycles (Lotka, 1925; Volterra, 1926), trophic cascades (Hairston et al., 1960), and dispersal (Fronhofer et al., 2018). In systems where consumers and resources are dynamically coupled by a strong interaction, the response of their densities to temperature change is interdependent on their individual thermal responses and their interaction. Both models (O'Connor, 2009; Vasseur & McCann, 2005) and experiments (Barneche et al., 2021) have shown that warming can negatively impact abundance across multiple trophic levels despite increasing rates of primary productivity.

In this paper, we consider how warming and resource densities impact the thermal niche and population dynamics of consumers in a pair of trophically‐coupled models of consumers and resources. We analyse the dynamical behaviour of these models across a range of temperatures and show how the consumer's thermal niche can be constructed as a realized thermal niche that accounts for the effects of temperature on resources. In the first scenario, resources are not directly impacted by temperature, but the thermal performance of the consumer generates indirect effects on resources via consumption. In the second, both resources and consumers depend directly on temperature, and indirect effects arise through their interaction. It is here that we find the greatest potential for the consumer's thermal niche to interact with resource densities to reshape the consumer's realized thermal niche. We also find that this scenario creates the necessary conditions for a metabolic meltdown to occur. Our work emphasizes the importance of the mismatch in thermal optimum and range between the consumer and its resource, and resource limitation as a key ingredient underlying the relationship between fundamental and realized thermal niches.

## MODEL DEVELOPMENT AND ANALYSIS

### Background

Temperature is well‐known to impact a number of vital processes underlying the ecology of population growth and species interactions (e.g. Brown et al., 2004). The first examples of trophodynamic models that included temperature as an independent variable relied on relationships derived from metabolic theory (Savage et al., 2004; Vasseur & McCann, 2005). Here Savage et al. and Vasseur and McCann assumed that the vital rates of resource production, resource consumption, and consumer respiration scaled according to the Boltzmann‐Arrhenius relationship but differed in their assumptions about the temperature‐dependence of resource carrying capacity. These models provided a useful framework to compare broadly across communities—consistent with the applications of metabolic theory—but they did not capture the dynamics inherent to individual communities where non‐linear (unimodal) responses can cause vital rates to decline when temperatures surpass $T_{\mathrm{opt}}$.

Amarasekare (2015) extended temperature‐dependent trophodynamic models to include unimodal responses to temperature in resource production and the parameters governing the type II consumer functional response. This work demonstrated that the $T_{\min}$ and $T_{\max}$ for the consumer population were jointly determined by resource and consumer response to temperature. Similar to how Liebig's law sets the rate of growth as a function of the most limiting resource, $T_{\min}$ and $T_{\max}$ can be determined by the thermal limitations of consumption or by the thermal limitations of resource growth. Furthermore, Amarasekare (2015) highlighted the potential for a thermal mismatch (differences amongst the optimum temperatures or shapes of the thermal responses of consumers and resources) to have significant impacts on dynamics. Note that what we define as a thermal mismatch is often described as ‘thermal asymmetry’ in the literature. The thermal mismatch is likely common in nature and could be exacerbated by climate change as new species interactions arise (Dell et al., 2014; Porter et al., 1973). The thermal mismatch can enter indirectly or directly into the temperature‐scaling of the functional response parameters (i.e. attack rate and handling time) (Dell et al., 2014). The temperature‐scaling of these parameters can be jointly, or individually determined by the resource or consumer's traits. A key challenge for incorporating thermal mismatch into trophodynamic models is understanding which model elements scale with temperature and how we might reasonably constrain the number and type of mismatches that can arise amongst the various model parameters (Amarasekare, 2015; Dell et al., 2014; Synodinos et al., 2021; Vasseur, 2020).

The unimodal temperature‐dependence of resource production, and the parameters *a* (attack rate) and *h* (handling time) of the type II functional response have been well established empirically (Bernhardt et al., 2018; Englund et al., 2011; Rall et al., 2010; Uszko et al., 2017); however, researchers have struggled to identify a general form for the temperature dependence of resource carrying capacity, K. The logistic model to which K belongs has been a mainstay in the study of population and community dynamics (e.g. Rosenzweig & MacArthur, 1963). However, there has been considerable debate about how to implement a temperature‐dependent version of the model due to the lack of empirical data on the temperature sensitivity of K (see Vasseur, 2020). Early attempts assumed that K was invariant (Vasseur & McCann, 2005) or that, due to the increased metabolic requirements of individuals in warmer temperatures, K would decline with warming (Savage et al., 2004). An empirical synthesis of the effects of temperature on model parameters found only four studies measuring the effect on K, and these unequivocally supported a decrease with warming—albeit over a restricted range of ‘biologically relevant’ temperatures (Fussmann et al., 2014). This result was also confirmed more recently by Bernhardt et al. (2018). More recent work (Lemoine, 2019) has stressed the non‐linear and often unimodal shapes of temperature dependence on biological rates as a reason to investigate the same functional dependence for K. Since K is the result of the joint expression of many biotic and abiotic processes, the manner in which these processes align can lead to a variety of different functional forms over the biologically relevant range (Lemoine, 2019; Uszko et al., 2017). As we endeavour to describe temperature's effect at the scale of communities, it is important to consider a broader range of temperatures, including those that could lead to negative rates of population growth. In fact, Vasseur (2020) recently demonstrated that doing so elucidates an important constraint on the functional form of K: that *r* and *K* must always share the same sign (positive/negative) as temperature (or any other driver) is varied (see below for more detail). In this paper, we utilize this constraint to advance our understanding of the impact of temperature on trophic communities.

BOX 1 **Why does $T_{\mathrm{opt}}$ shift to lower temperatures under resource limitation**

Brett et al. (1969) and Brett (1971) experimentally demonstrated that the optimal temperature for individual growth shifted to cooler temperatures under resource limitation and the mechanistic basis for this result has been shown to rely upon differences in the non‐linear functions determining the positive and negative contributions of net energy gain to growth (Huey & Kingsolver, 2019). Thomas et al. (2017) demonstrated a similar shift in the optimal temperature for algal population growth under abiotic nutrient limitation highlighting that this outcome occurs at both the individual and population levels. Here we show how such a shift can arise for a heterotrophic consumer and we introduce the framework and assumptions upon which our modelling work is based.

The rate of accumulation of biomass by a heterotrophic population (C) is proportional to the rate at which it assimilates consumed biomass, less the amount of biomass that is respired in order to maintain vital functions (Yodzis & Innes, 1992):

(1) $$\frac{\mathrm{dC}}{\mathrm{Cdt}}=\left(1-\delta \right)f\left(R,T\right)-m\left(T\right)$$

Here, *C* is given in units of population biomass rather than numbers of individuals. $\left(1-\delta \right)$ is the fraction of consumed biomass that is assimilated, $f\left(R,T\right)$ is the functional response and m represents the rate of respiration. It is well known that both the consumption and respiration terms of Equation 1 are sensitive to temperature (*T*) and most interpretations of consumer growth assume that only the functional response is influenced by resource density (R) (but see DeLong & Vasseur, 2012; Ginzburg & Akcakaya, 1992). Under these assumptions, if there is a value of T for which dC/Cdt is maximized (i.e. if a thermal optimum exists), and $f\left(R,T\right)$ is an increasing function of R, then the temperature that maximizes dC/Cdtwill increase with resource density.

Following empirical evidence, it is commonly assumed that for an ectothermic consumer the respiration rate m rises with temperature according to a Boltzmann‐Arrhenius relationship (Brown et al., 2004; Gillooly et al., 2007; Vasseur & McCann, 2005). We approximate this relationship with a simple exponential function:

(2) $$m\left(T\right)=m_{a}e^{m_{b}T}+m_{c}$$

where $m_{a}$, $m_{b}$ and $m_{c}>0$. The functional response has been shown to exhibit temperature dependence under a wide range of conditions (Daugaard et al., 2019; Englund et al., 2011; Rall et al., 2010; Uszko et al., 2017). We employ type II, where the attack rate (a) and handling rate (1/h) have been shown to exhibit a hump‐shaped relationship with temperature (Englund et al., 2011). We use the Michaelis–Menten form of the type II functional response:

(3) $$f\left(R,T\right)=I_{\max}\left(T\right)∙\frac{R}{R+R_{0}}$$

where $I_{\max}\left(T\right)$ is the maximum uptake rate and $R_{0}$ is the half‐saturation density. This form relates to the Holling Type II form via two equivalencies: $I_{\max}\left(T\right)\equiv 1/h$ and $R_{0}\equiv 1/\left(a∙h\right)$, where *h* is the handling time and *a* is the attack rate. We assume that $R_{0}$ is temperature‐invariant due to the fact that a and1/h exhibit similar responses to temperature (Englund et al., 2011; Vasseur & McCann, 2005). This form allows us to isolate the extent of resource saturation, $R/\left(R_{0}+R\right)$, independently of the effect of temperature on $I_{\max}$. Here, we follow previous work which assumed a symmetric unimodal relationship with temperature (Amarasekare, 2015; Amarasekare & Savage, 2012):

(4) $$I_{\max}\left(T\right)=e^{-{\left(T-T_{I}\right)}^{2}/\beta }$$

where $T_{I}$ gives the optimum temperature for consumption (note that this is different from $T_{\mathrm{opt}}$ for the TPC) and β is a parameter determining the breadth of the response. Although the temperature dependence of ingestion may be best described by an asymmetric unimodal function (Dell et al., 2011) the use of a symmetric form does not change the qualitative nature of our results.

**FIGURE 1:**
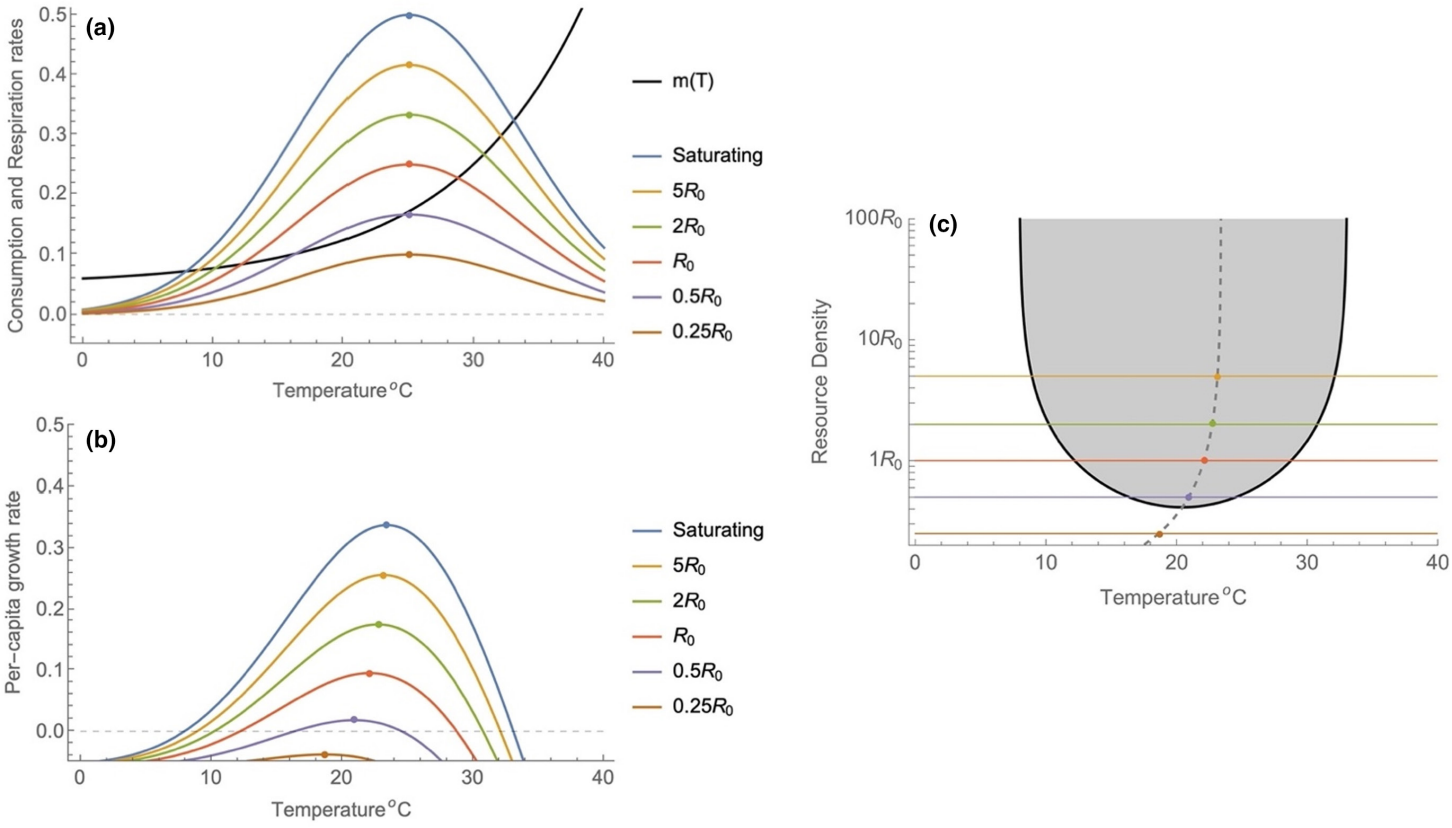
Non‐linear effects of temperature on consumption and respiration rates interact with resource density to shift the optimum temperature for population growth. The colours of the lines represent consumption at different resource availabilities, and $m\left(T\right)$ represents the rate of respiration. Panel a) shows a family of curves representing different resource densities (scaled relative to the half‐saturation rate) and the respiration rate of a consumer. The difference between each consumption curve and the respiration curve gives the per‐capita growth rate (panel b) and shows a decline in the temperature which maximizes per‐capita growth. As the density of resources declines, the ingestion rate decreases, leading to changes in the intercepts and optimum of the TPC; the lower threshold temperature ($T_{\min}$) and upper threshold temperature ($T_{\max}$) converge under resource limitation and at the same time the optimum shifts toward lower temperatures due to the interplay of the non‐linear functions governing ingestion and maintenance. In panel c, the filled area represents combinations of temperature and resource density that generate a positive per‐unit growth rate and the black line represents the zero‐net‐growth‐isocline of the consumer. This isocline can be separated into the lower and upper intercepts of the thermal performance curves in panel b. this isocline is nearly symmetric about minimum, reflecting the strong impact of resources on I_max_. The optimum temperature for growth at each resource density is given by the dotted line in panel c. here =25, β=150, =0.01, =0.1, =0.05, and δ=0.5.

Taken together, these assumptions generate a shift in the optimal temperature for population growth as resource densities change (Box Figure 1b). Under resource saturated conditions, the optimal temperature for growth approaches the thermal optimum of the consumption function (T_I_); however, as resources decline, the optimal temperature for growth shifts to cooler temperatures. The temperatures at which the consumption and respiration rates intersect represent the lower and upper critical temperatures for population growth (*T*_*min*_ and *T*_*max*_); these limits are shown in Box Figure 1c where they trace the consumer's niche as a function of temperature and resource densities. The consumer's niche is bounded by $T_{\min}$ (on the colder side) and $T_{\max}$ (on the warmer side) and at the point where they connect (the limit where the TPC collapses to a single point), positive per‐unit growth of the consumer can occur only at the temperature that is midway between the $T_{\min}$ and $T_{\max}$. If we use the formalism of $R^{*}$theory (Tilman, 1977), it means that the consumer's $R^{*}$, or the lowest level of limiting resource, is midway between $T_{\min}$ and $T_{\max}$, not at $T_{\mathrm{opt}}$. Thomas et al. (2017) demonstrated this same outcome using a ‘double‐exponential’ model where algal production (photosynthesis) and respiration both scaled exponentially (but differently) with temperature. It is also noteworthy that the assimilation fraction δ can have a similar effect on resource density. Assuming that the assimilation efficiency is not temperature sensitive and there is experimental evidence both for and against this (Alexander et al., 2012; Xu & Ji, 2006), declining efficiency due to changes in resource quality will have the same effect on thermal performance.

### Dynamic Consumer‐Resource models

Below we couple the consumption theory described in Box 1 with two different models of resource dynamics to consider how the dynamic interplay between resources and consumers determines a consumer's realized TPC. We describe first a model where resources are supplied in a chemostat with supply concentrations and flow that are not sensitive to temperature. This model allows us to investigate the role of feedback in the absence of any direct effect of temperature on resources. We then analyse a second model where we assume a biotic resource growing logistically and has temperature sensitivity.

### Model 1: Chemostat model

We begin our analysis of temperature‐dependent consumer‐resource dynamics using a chemostat model of resource supply, where resources are assumed to be non‐living nutrients. Here, resources are provided at an inflow density S and flow rate D and neither parameter are sensitive to temperature. Combined with the resource functional response (Equation 3), the equation for resource dynamics is given by:

(5) $$\frac{\mathrm{dR}}{\mathrm{dt}}=D\left(S-R\right)-f\left(R,T\right)C$$

together with Equations (1), (2), (3), (4), this generates an ordinary differential equation system that exhibits two possible equilibria solutions ($\xi^{R,C}$) corresponding to a resource‐only equilibrium ($\xi^{+,0}$) and a mixed consumer‐resource equilibrium ($\xi^{+,+}$). These equilibrium solutions and their stability can be solved analytically; we provide these in Appendix A1. For this model, one of the two possible equilibria is always stable (except at the transcritical bifurcation points).

**FIGURE 2:**
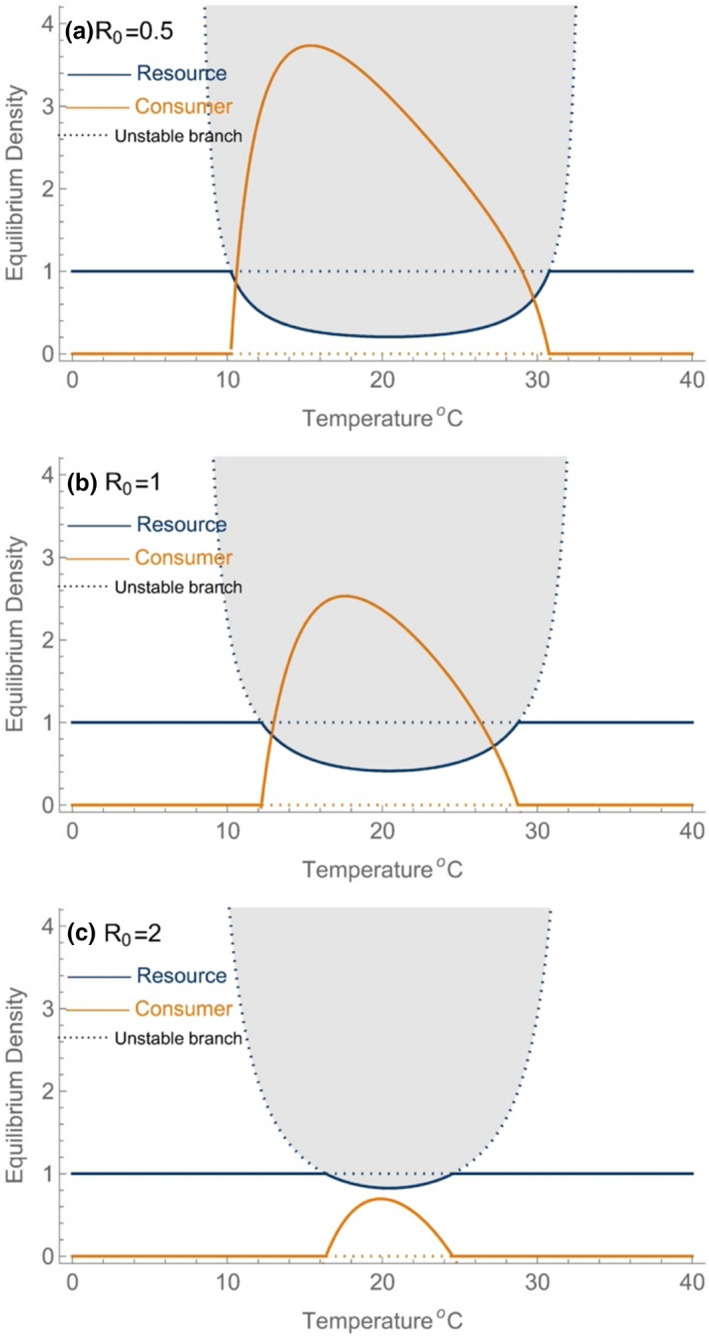
Equilibrium densities of the consumer‐resource dynamic model when resources are supplied by a temperature‐independent chemostat for an efficient (a) and prudent (b) consumer. Here solid (dotted) lines correspond to stable (unstable) equilibria. We have omitted branches some unstable branches for clarity. Transcritical bifurcations occur where the two equilibrium branches exchange stability (e.g. at approximately 10 and 30.5 °C in panel a). The U‐shaped equilibrium branch for the resource population (filled in grey) is a rescaled version of the curve shown in Box Figure 1c. Higher values of R_0_ correspond to more prudent consumers which require higher resource density to achieve the same saturation of the functional response. The equilibrium densities are analytically solved in Appendix A1. Parameter values are as in Box Figure 1 and S=D=1.

When the combination of the resource supply concentration (S) and temperature (T) falls outside the range allowing positive consumer growth (the shaded envelope defined in Box Figure 1c), the resource‐only equilibrium is stable and is unchanged by temperature (Figure 2). This occurs at both low‐ and high‐temperature combinations. At temperatures where the resource supply combination and temperature permit consumer growth, resources are consumed to an equilibrium concentration at which the per‐unit rate of consumer growth, dC/Cdt, is equal to zero. This is the curve tracing $T_{\min}$ and $T_{\max}$ in Box Figure 1c and is shown again by the resource equilibrium in Figure 2.

**FIGURE A1:**
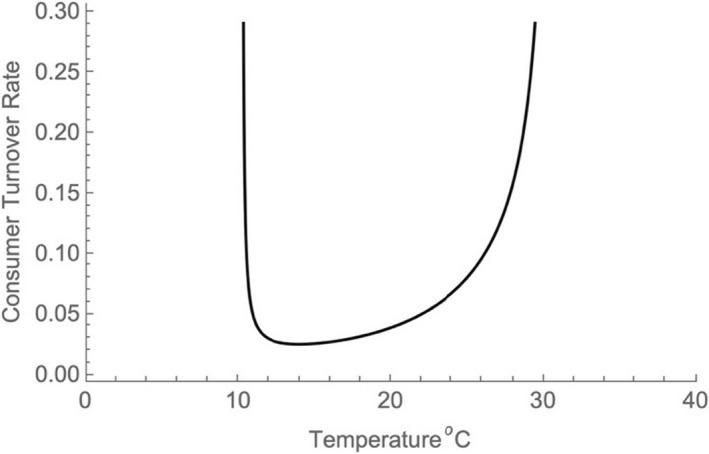
Consumer biomass turnover rate for the chemostat‐resource model. Turnover is given by the ratio between consumer production and equilibrium biomass. This ratio has vertical asymptotes where the consumer equilibrium reaches its lower and upper critical values. Away from these asymptotes, the turnover rate increases with temperature reflecting an increase in biomass expenditure for consumer respiration per unit of consumed biomass.

**FIGURE A2:**
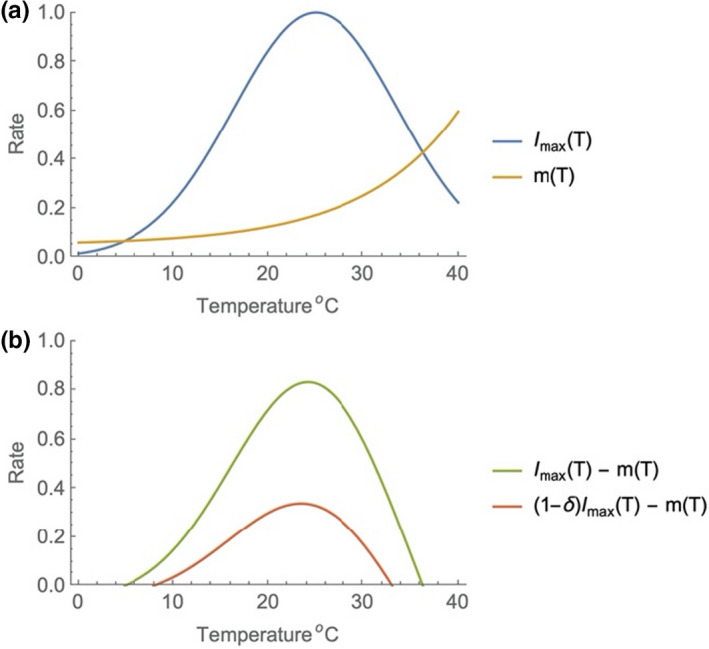
a) Changes in $I_{\max}$ (blue) and m (yellow) across temperature and b) changes in the difference between $I_{\max}$ and m as a function of temperature (green) and when reduced by the assimilated fraction (1‐ δ) (orange). The difference amongst the two non‐linear functions in panel a results in the skewed unimodal functions in panel b that are typical of thermal performance curves. The intercepts of the functions in panel b are the critical points, respectively. Here, $T_{I}=25$, β=150, $m_{a}=0.01$, $m_{b}=0.1$, $m_{c}=0.05$, and δ=0.5.

The equilibrium dynamic for the consumer population is an asymmetric unimodal function which has a skewness that is opposite to the TPC, peaking at values nearer to $T_{\min}$ than $T_{\max}$ (Figure 2). An important contributor to this difference in skewness and location of the optimum is the greater rate of consumer biomass turnover occurring at warmer temperatures (Figure A1). Turnover, measured as production/biomass is a U‐shaped function with asymptotes at $T_{\min}$ and $T_{\max}$ (due to the fact that here, equilibrium biomass →0). Turnover is lowest at temperatures near $T_{\min}$ and then increases approximately exponentially with warming. With increases in turnover, more biomass is lost to conversion inefficiency. Coupled with higher rates of metabolism at warmer temperatures, this leads to a peak in the equilibrium consumer biomass peaks at temperatures below $T_{I}$ and gradual declines in equilibrium consumer biomass with warming (Figure A2).

Under this set of assumptions, $T_{\max}$ and $T_{\min}$ are determined by the consumer vital rates, since the supply of resources is independent of temperature. As consumers approach their upper or lower thermal limits, their population size declines and top‐down control of resources relaxes, allowing resource equilibrium densities to increase (Figure 2). In this scenario, resource densities always increase as consumers approach *T*_max_ because the impact of temperature on resource densities is an indirect effect and resource density is entirely under top‐down control. Even when top‐down control is relaxed by making the consumer more prudent, here increasing $R_{0}$ (Figure 2b), resource densities increase as consumers approach their upper thermal limit. Adjusting $R_{0}$ allows for a change in the ‘perceived’ resource density for the consumer, and although altering S and D will also impact resource density in a qualitatively similar way, a change in $R_{0}$ allows the comparison with the following consumer‐resource model with logistic growth.

### Consumer‐resource model with logistic growth

The logistic growth model can be easily derived by assuming density‐dependence reduces the rate of per‐unit birth or increases the per‐unit rate of death in a population. The parameters *r* and *K* express the density‐independent rate of per‐unit growth and joint effects of density dependence on birth and death respectively. The impact of temperature on the per‐unit rate of population growth is well‐defined for both positive and negative values. Whilst there has been some debate on how best to incorporate these negative values in forecasting risk (Woods et al., 2018), negative rates of population growth have an important role in understanding transient behaviour. The incorporation of negative intrinsic growth rates into the logistic model is challenged by the fact that a transition to negative values leads to an inversion of the effect of density dependence. To see this, consider the sign change of the second term in $r\left(1-\frac{N}{K}\right)$ when r transitions from positive to negative values. The effect of this sign change is that density dependence contributes beneficially to population growth during periods of decline, when in fact, density dependence should continue to have a negative effect.

Mallet (2012) suggested an alternative framework for the logistic model (the r−α model) to cope with the issue of negative population growth. In this model, the effects of *r* are separated from the density‐dependent parameter α, allowing a sign change in *r* to propagate properly into population dynamics. However, (Vasseur, 2020) showed that it is unnecessary to switch to an alternative framework when the appropriate constraints are considered. Vasseur demonstrated that when temperature or another environmental variable causes *r* to switch from positive to negative value, K must also switch from positive to negative value. Although prescribing a negative carrying capacity may seem illogical, it is important to note that negative carrying capacities never constitute stable equilibrium points in the Logistic model, but instead accurately guide the population dynamics to extinction during periods of stress. The postulation that r and K must have the same sign, also requires that they have the same intercepts. Combined with our confidence in the form of temperature dependence of r, this observation allows us to assume that K may be, in its simplest form, a unimodal function of temperature that is positive across the domain $T_{\min}$ to $T_{\max}$. There have been relatively few experimental attempts to determine the impact of temperature on carrying capacity and these have shown mixed results including monotonic decreases, independence and unimodality (Bernhardt et al., 2018; Fussmann et al., 2014). The extent to which K can differ from r, within the bounds of these constraints, has been explored only to a limited extent mathematically (see Vasseur, 2020).

Given these constraints, we model the rate of change of a logistically growing resource with a consumer is given by:

(6) $$\frac{\mathrm{dR}}{\mathrm{dt}}=r\left(T\right)R\left[1-\frac{R}{K\left(T\right)}\right]-f\left(R,T\right)C$$

We generate a temperature‐dependent intrinsic rate of resource growth as the difference between a symmetric unimodal “growth” function and an exponentially increasing “maintenance” function similar to the assumptions governing our consumer in Equations 3 and 4:

(7) $$r\left(T\right)=b_{\max}e^{-{\left(T-T_{I}-{∆}_{T}\right)}^{2}/\beta }-\left(d_{0}+d_{1}\;e^{d_{2}T}\right)$$

Here, we use the same optimum, $T_{I}$, as specified for the consumer's ingestion rate (Equation 3); however, we add the parameter ${∆}_{T}$ to represent the potential for a thermal mismatch to arise via a shift in the optimum temperature for resource growth relative to the consumer. The temperature‐dependent carrying capacity $K\left(T\right)$ is given by:

(8) $$K\left(T\right)=\frac{r\left(T\right)}{\gamma }$$

where γ represents the joint effects of density‐dependence on birth and death of the resource population. Here we do not assume temperature effects the strength of density‐dependence (i.e. if temperature altered the impact of population size on birth or death); however, Vasseur (2020) showed that if warming strengthens density‐dependence, $K\left(T\right)$ peaks at a lower temperature than $r\left(T\right)$ (similar to the patterns shown for the consumer in the chemostat model).

This model has three possible equilibrium states: a trivial equilibrium ($\xi^{0,0}$), a resource‐only equilibrium ($\xi^{+,0}$), and a mixed equilibrium ($\xi^{+,+}$). Analytical solutions for these equilibria and their stability are provided in Appendix A1. In contrast to the chemostat model, this model can also produce a mixed equilibrium point which is unstable, resulting in a stable limit cycle.

**FIGURE 3:**
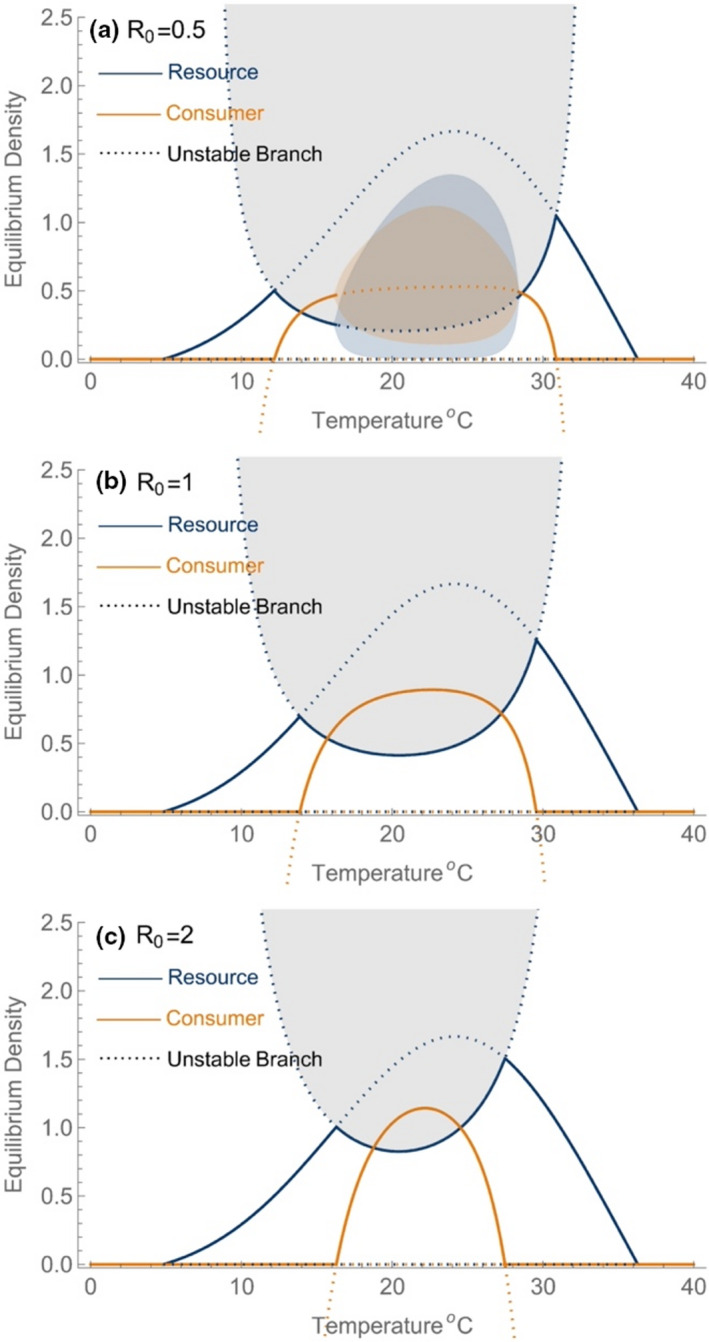
The dynamics of the consumer and resource when the resource's dynamics are given by the logistic model with temperature‐sensitive parameters r and K for three values of R_0_ (0.5, 1.0 and 2.0). Stable equilibrium branches are shown as solid lines and unstable branches as dashed lines. We have omitted some unstable branches for clarity. The exchange of stability between equilibrium branches ($\xi^{i}$) occurs at transcritical bifurcations. The coexistence equilibrium ($\xi^{+,+}$) loses stability (and regains) stability at Hopf bifurcation points which generate limit cycles (with amplitudes shown by the shaded regions). The U‐shaped equilibrium branch for the resource population (filled in grey) is a rescaled version of the curve shown in Box Figure 1c. Higher values of R_0_ correspond to more prudent consumers which require higher resource density to achieve the same saturation of the functional response. Parameters are: 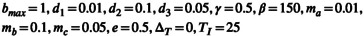.

Figure 3 shows the dynamics of the model using three sample parameter sets where no thermal mismatch exists amongst the resource and consumer and where the resource has a wider fundamental thermal niche than the consumer. As temperature increases from low values, the system transitions from the trivial equilibrium to the resource‐only equilibrium that tracks $K\left(T\right)$ until the combination of temperature and resource density permit consumer growth. Here, the community transitions to the stable mixed equilibrium but with further warming, it may undergo a Hopf bifurcation to limit cycles depending on the strength of top‐down control. As the system approaches upper limiting temperatures, this sequence of events is reversed. In this scenario, we demonstrate that again, resource densities increase as temperatures approach the limiting value for consumer persistence ($T_{\max}$). Despite the fact that the resource‐only equilibrium also declines as the consumer approaches its thermal limits, the increase in resource densities caused by a relaxation of top‐down control is superseding factor. Even when consumers are extremely prudent (Figure 3c) resources increase as consumers decline.

**FIGURE 4:**
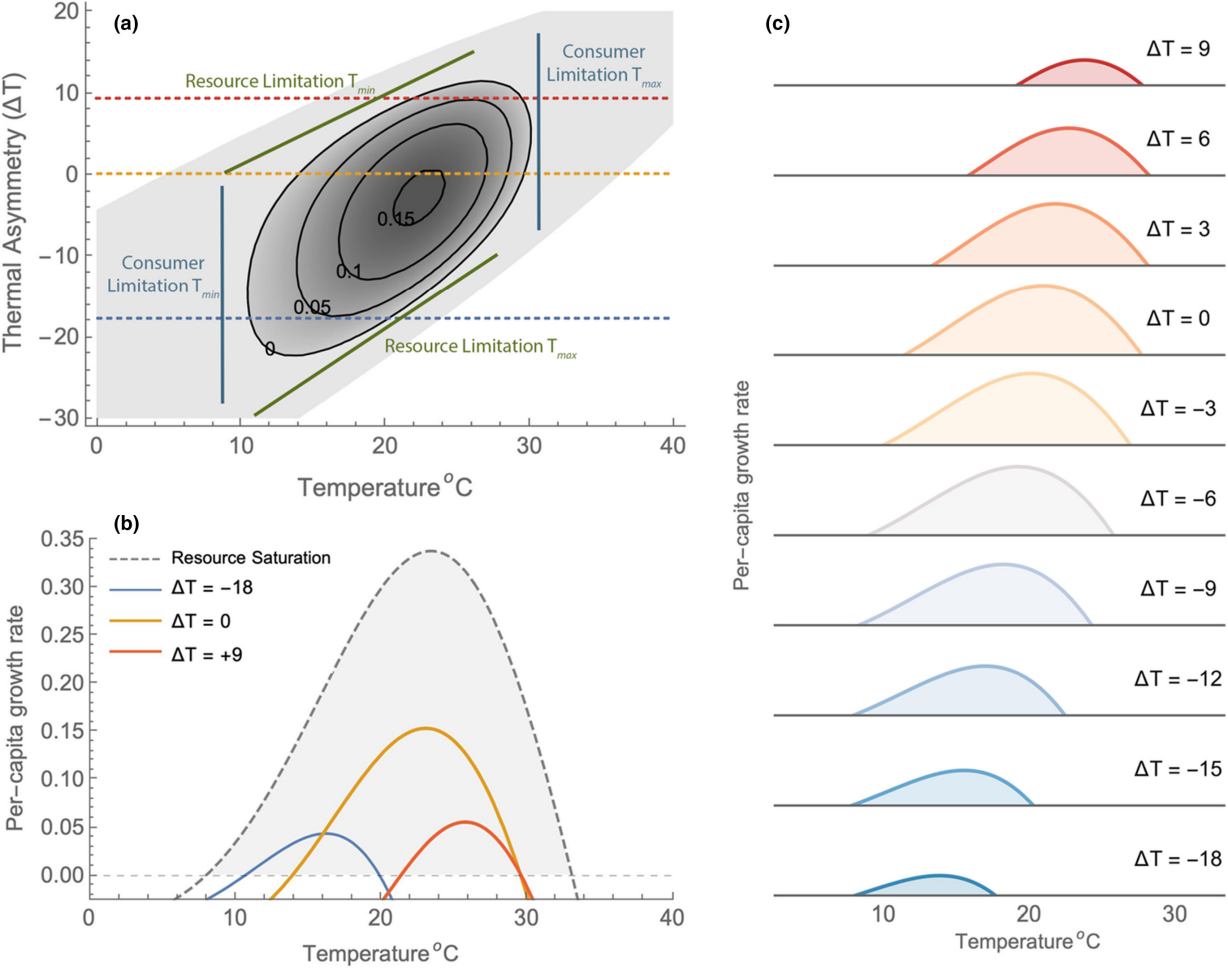
The effect of thermal asymmetry on the dynamics of consumers and resources for logistic resource model with $R_{0}=1$. Panel a) the shaded areas depict which of the dynamical regimes is the stable outcome for each combination of temperature and thermal asymmetry (positive values shift the resource's thermal performance to warmer temperatures). In the unshaded area, neither resource nor consumer can persist ($\xi^{0,0}$); in the lightly shaded area only the resource persists ($\xi^{+,0}$); in the darkest region resources and consumers persist at a stable equilibrium ($\xi^{+,+}$). No limit cycles emerge for these parameter values. For negative asymmetry, resources limit $T_{\max}$ whereas consumption limits $T_{\min}$; for positive asymmetry, the results are reversed. Panel b) shows the realized thermal performance curves for three values of thermal asymmetry and panel c) shows the full range of thermal performance curves that are generated as thermal asymmetry varies. Parameter values are given in Figure 3.

We assess the importance of thermal mismatch on the thermal performance and dynamics of the consumer by adjusting the optimum of the resource's “growth” function relative to the optimum for consumer ingestion ($T_{\mathrm{opt}}$). This parameter (${∆}_{T}$) shifts both the $r\left(T\right)$ and $K\left(T\right)$ functions in accordance with Equations 7 and 8 and, therefore, moves the envelope of temperatures over which the resource can achieve a positive equilibrium density (Figure 4). This has important effects on the consumer's upper and lower thermal tolerances for growth. As the resource's thermal performance shifts to warmer temperatures (positive ${∆}_{T})$, the consumer's $T_{\min}$ increases to reflect the effect of resource limitation at low temperatures due to the resource's temperature dependence. Similarly, if the resource's thermal performance shifts to colder temperatures, the consumer's $T_{\max}$ becomes limited by the resource's thermal performance and shifts to lower temperatures (Figure 4).

**FIGURE 5:**
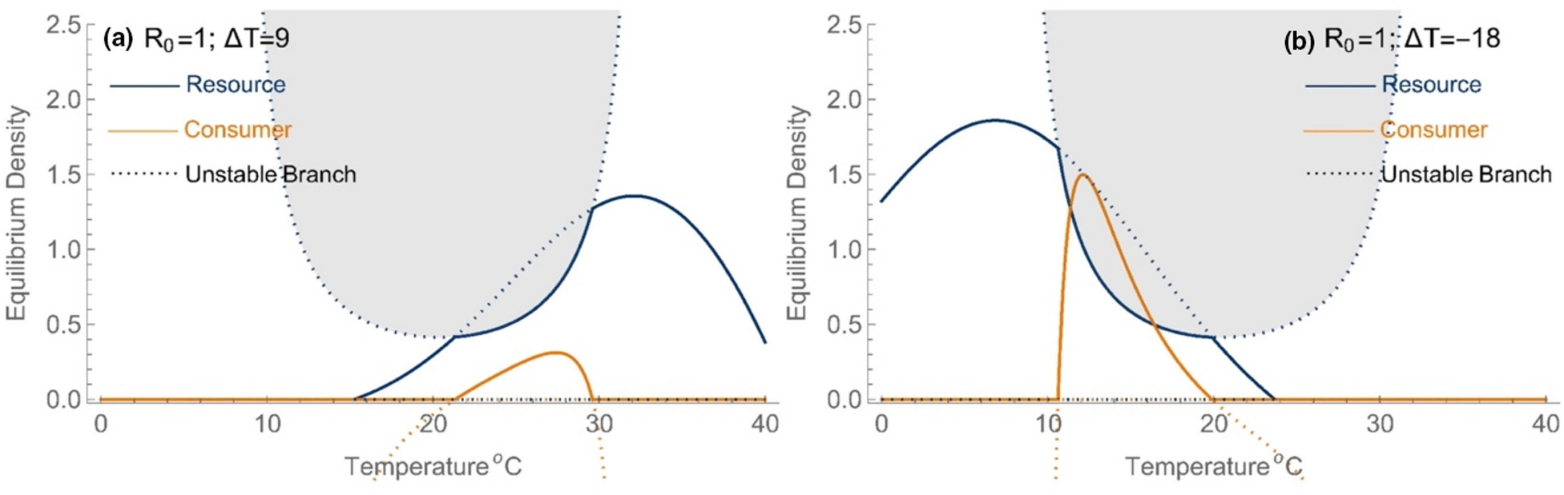
Panels (a) and (b) show traces of the resource equilibria across temperatures for the scenarios shown in Figure 4 for (a) positive thermal asymmetry ($∆T=+9^{{}^{\circ}}C$) and (b) negative thermal asymmetry ($∆T=-18^{{}^{\circ}}C$). See Figure 2 for details on curves. For resource densities to continue to decline as consumers approach their thermal limits the upper (lower) limit must fall to the left (right) of the minimum of the consumer's thermal niche, which is given by the shaded region. These scenarios highlight the differences in the resource dynamics when consumer persistence is limited by temperature (Figure 3) vs. when it is limited by resources. Parameter values are as in Figure 3

The effect of thermal mismatch on the realized thermal performance of consumers and resources and their dynamics are shown in Figure 5. When a thermal mismatch is large enough that resource thermal traits (r and K) determine the consumer's $T_{\min}$ or $T_{\max}$, we find scenarios where temperature‐driven declines in resource density are not superseded by a relaxation in top‐down control. Specifically, when the contours of the resource‐only equilibrium and the consumer's zero‐net‐growth isocline (ZNGI) intersect so that $T_{\max}$ is located on a negative‐sloping region of the ZNGI, resource densities will decline with warming as consumer's approach $T_{\max}$—consistent with the conditions necessary for a ‘metabolic meltdown’ (Figure 5). The same observation is true for a cooling effect if $T_{\min}$ is located on a positive‐sloping region of the ZNGI, although would not be associated with a metabolic meltdown. In these two scenarios, the consumer's realized thermal performance curves are greatly re‐shaped by the resource's response to temperature in the absence of consumption (r and K; Figure 4b,c). We demonstrate the variation in the consumer's thermal performance curves that can be generated by the mismatch of thermal responses in Figure 4c.

## DISCUSSION

**FIGURE A3:**
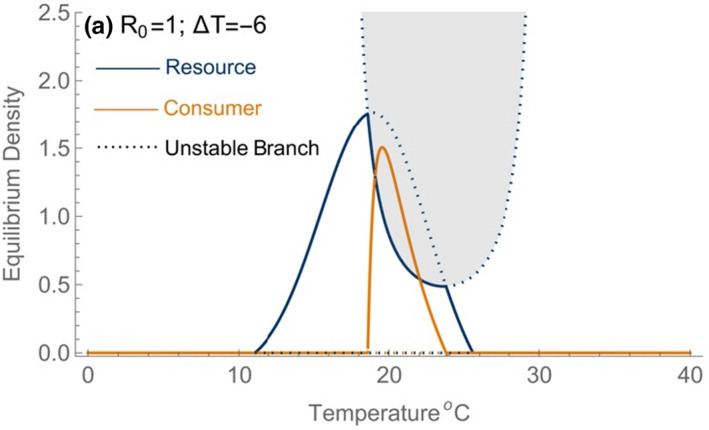
Traces of the equilibrium densities of consumers and resources with negative thermal asymmetry ($∆T=-6^{{}^{\circ}}C$) but where the breadth of thermal performance is narrower compared to Figure 4. Parameter values are as in Figure 3 but β for resource growth is 25 (Equation 7) and β for consumer growth is 40 (Equation 3).

Hutchinson defined the niche as the set of all environments in which the density‐independent rate of birth exceeds the rate of death, or more simply those environments in which r≥0 (Holt, 2009). Here, we show that the TPC, when defined in the context of r, exhibits important non‐linear interactions with resource density. In particular, the ‘realized’ thermal performance of the consumer and resource can be drastically altered relative to the ‘fundamental’ (density‐independent) thermal performance. The observation that both the optimum and breadth of the consumer realized performance curves shift to cooler temperatures as resource densities decline suggests that warming, when coupled with a decline in resources could exacerbate extinction risk and generate the necessary conditions for a metabolic meltdown (Huey & Kingsolver, 2019). Our model only produces this outcome when a thermal mismatch exists between resources and consumers. When a mismatch is small, the relaxation of strong top‐down control that occurs as consumers approach the limits of their realized thermal niche allows resources to rebound to larger values. When a mismatch is large and the limits of the consumer's thermal niche are determined by the resource's thermal traits (rather than by its own physiological limits), top‐down control is weak and metabolic meltdown is more likely to occur. Although this requires a substantial amount of mismatch (Figures 4 and 5), the same result can be achieved with a much smaller difference in thermal optima provided that the breadth of thermal performance curves also differs amongst consumers and resources (see Figure A3). Barbier and Loreau (2019) demonstrated that there is wide variation in the extent to which strong top‐down control exists, and structures food chains. Typically, this depends upon the strength of self‐regulation of populations but here we demonstrate, in the absence of consumer self‐regulation, that temperature can limit consumer populations, weaken top‐down control and allow resource densities to increase as consumers approach their thermal limits. However, communities that are inherently bottom‐up regulated, such as those under donor control (Strong, 1992) or those driven by a resource subsidy, could exhibit a reduction in resource densities coincidentally with warming without the need for a thermal mismatch.

Mismatch amongst the responses of consumers and resources has been a subject of recent interest (Amarasekare, 2015; Dell et al., 2014; Smith & Amarasekare, 2018) particularly given that responses to environmental change may be more rapid for some species relative to others. Whilst we define mismatch in our model as a difference between the consumer and resource optimal values of the functions representing energy intake, mismatch may be more important with respect to differences amongst $T_{\min}$ and $T_{\max}$ (which our model consequently generates). Dell et al. (2011) found that prey tends to have lower physiological minima (${\mathrm{CT}}_{\min}$) than their predators and suggested that prey is under selection for broader thermal performance curves to avoid being eaten (life‐dinner principle). Similarly, others have shown that prey has a higher ${\mathrm{CT}}_{\max}$ than their predators (Petchey et al., 1999; Sentis et al., 2013; Voigt et al., 2003), but see (Franken et al., 2018). Lancaster and Humphreys (2020) showed that land plants have very wide thermal tolerances, suggesting that herbivores are unlikely to be influenced by reductions in resources as they near *T*_*max*_ unless other environmental changes occur in concert. In host‐pathogen interactions, the infection rate can increase with host/parasite thermal tolerance mismatch (Nowakowski et al., 2016). Parasites tend to have wider tolerance (lower $T_{\min}$ and higher $T_{\max}$) than hosts, so hosts become more at risk when temperatures shift outside of their limits (Cohen et al., 2017). Moreover, whilst our model assumes sessile resources, such that the capture rate is entirely consumer‐trait driven, motile resources (i.e. prey) may become easier to catch as they near ${\mathrm{CT}}_{\min}$ and ${\mathrm{CT}}_{\max}$. Traits such as palatability and size of the resource may play a role in structuring these relationships. Redefining our model to include a more accurate depiction of the capture rate (e.g. Dell et al., 2014; Pawar et al., 2012) may provide greater insight into the broader importance of thermal mismatch.

Amarasekare (2015) analysed consumer resource models under a variety of different assumptions about the temperature dependence of vital rates. Although Amarasekare (2015) similarly found that consumer persistence critically depends upon resource thermal traits, they explored these dynamics under the assumptions of either monotonically increasing or U‐shaped temperature dependence of carrying capacity. Although the latter case limits the thermal performance of resources to a finite temperature range, thereby allowing resource thermal traits to impact a consumer's thermal performance curve, it may also weaken top‐down control of resources due to a relaxation of resource density dependence. Furthermore, these assumptions violate the requirement that r and K must have the same sign in order for density‐dependence to be correctly incorporated (Vasseur, 2020).

Our models consider only a focal pair of consumers and resources; however few systems are isolated from the effects of interactions with additional species (Gilman et al., 2010; Kordas et al., 2011). In instances where more than one species impacts resource densities, a reduction in resource densities with warming, and the potential for metabolic meltdown may be far more likely than we predict. For example, when one or more other competing consumers share a resource, resource densities become increasingly under the control of competitors as the focal consumer nears its $T_{\max}\;$(and competitors do not). Temperature change has been shown to reduce vertical niche segregation in deep cold lakes of species including whitefish and vendace (Gjelland et al., 2007). These fish both feed on zooplankton, and thus the increasing spatial overlap between these predators will likely lead to local depletion in zooplankton and could put the species with lower $T_{\max}$ at an increased risk. Furthermore, the change in top‐down control of resources may be particularly prevalent in cases where invasive species are better suited to warmer conditions outside the range of a focal consumer (Finch et al., 2021). Such ‘community level’ effects could also extend to cases where abiotic resources do not exhibit direct responses to temperature (e.g. our chemostat model); instead, thermal mismatch amongst competitors could alter resource productivity and availability in colder or warmer temperatures, mimicking the effects of thermal mismatch that we describe for the logistic model.

In our logistic resource model, we assume that $r\left(T\right)$ and $K\left(T\right)$ respond symmetrically to temperature—which implies that density dependence in the resource population is not affected by temperature (but see Vasseur, 2020). Resource populations may be under similar constraints as those that generate the differences in the productivity and equilibrium biomass of consumers in our model. This would shift the optimal value of K(T) to cooler temperatures relative to $r\left(T\right)$, and enhance consumer growth at cooler temperatures. Further work should aim to improve our understanding of how biotic and abiotic resource populations' response to temperature can best be described in our models. We refrain from conducting a full analysis of parameter sensitivity in this paper because the consumer‐resource frameworks that form the backbone of our models confine their outcomes to a set of previously well‐studied regimes (e.g. stable equilibria, cycles) where the effects of parameter changes are well‐known (see Yodzis & Innes, 1992). Here, we highlight how changing temperature selects amongst these well‐studied regimes.

Although our models build on previous work to incorporate temperature‐rate relationships and species interactions in concert, these dependencies may have important effects beyond the impacts included here. Resource scarcity may further impact the shape of the thermal performance curve if the metabolic rate is reduced during starvation (Auer et al., 2015) or incorporates the cost of digestion. Moreover, our models do not incorporate the well‐known effects of acclimation or plasticity in response to changing temperatures. Organisms can acclimate to temperature change via changes in metabolic rates and functional response parameters (Seebacher et al., 2015; Sentis & Morisson, 2015; Sohlström et al., 2021); however, this acclimation to temperature can be a complicated mix of beneficial and non‐beneficial responses (Aranguren‐Gassis et al. 2019). As temperature change pushes consumers toward their persistence boundary, acclismation may, therefore, buffer or exacerbate the risk of extinction in the short term; however, over a longer time scales it would be unlikely for acclimation to interfere with the joint effects of temperature and resource density we describe in this paper.

This study builds upon the work of Thomas et al. (2017) and Huey and Kingsolver (2019) and demonstrates that, via their influence on resource densities, a consumers' realized thermal niche can be substantially different than its fundamental thermal niche—or what is commonly called the TPC. The differences can be further exacerbated when resources are themselves directly affected by temperature. Operationally, we are far from being able to make predictions using realized TPCs, particularly where new relationships amongst herbivores and plants or predators and prey might emerge with climate change. However, our framework makes the straightforward prediction that consumer $T_{\max}$ and $T_{\min}$ both change in response to resource densities; where we expect resources to become more limiting, thermal safety margins will need to be greater to ensure that consumers persist. In resource‐fed systems such as rivers and lakes, our chemostat model demonstrates that nutrient loading from anthropogenic sources may actually increase the range of temperatures over which consumers (e.g. algae and bacteria) can achieve population growth. A better understanding of this phenomenon may help identify the causes of nuisance or toxic algal blooms and identify opportunities for mitigation. Overall, this generalizable framework where we develop a mechanistic link between the role of temperature change, species interactions and persistence both identifies gaps in our understanding of thermal responses, and takes a vital step toward improving forecasts of community response to climate change.

## AUTHORSHIP

ACV and DAV designed the study and conducted data analysis. ACV wrote the manuscript with substantial input from DAV.

## FUNDING INFORMATION

ACV acknowledges the support of an NSF PRFB #2010783, Yale University, and the University of Oxford. DAV acknowledges the support of NSF DEB #1754012 and Yale University. NSF DEB, Grant/Award Number: 1856279.

### PEER REVIEW

The peer review history for this article is available at https://publons.com/publon/10.1111/ele.14086.

## Data Availability

The equations and code to produce this manuscript are available on Zenodo with DOI 10.5281/zenodo.6818405.

## APPENDIX A

### A.1 Equilibrium solutions for the consumer‐resource models

#### A.1.1. Chemostat model

The consumer‐resource model, assuming chemostat resource renewal is given by:

$$\frac{\mathrm{dC}}{\mathrm{dt}}=\left(1-\delta \right)f\left(R,T\right)∙C-m\left(T\right)∙C$$

$$\frac{\mathrm{dR}}{\mathrm{dt}}=D\left(S-R\right)-f\left(R,T\right)∙C$$

where

$$f\left(R,T\right)=I_{\max}\left(T\right)∙\frac{R}{R+R_{0}}$$

This model has two equilibrium solutions, and we categorize them based on the presence or absence of the consumer and resource:

$$\text{Resource only}\;\left(\xi^{+,0}\right):R_{e}=S\;\text{and}\;C_{e}=0$$

$$\text{Coexistence}\;\left(\xi^{+,+}\right):R_{e}=\frac{m\;R_{0}}{\left(1-\delta \right)I_{\max}\left(T\right)-m}\;\text{and}$$

$$\;C_{e}=\frac{D\left(S-R_{e}\right)\left(R_{e}+R_{0}\right)}{I_{\max}\left(T\right)\;R_{e}}$$

Note that for coexistence, the consumer's equilibrium is written as a function of the resource equilibrium *R*_*e*_.

#### A.1.2. Logistic model

The consumer‐resource model, assuming Logistic resource renewal is given by:

$$\frac{\mathrm{dC}}{\mathrm{dt}}=\left(1-\delta \right)f\left(R,T\right)∙C-m\left(T\right)∙C$$

$$\frac{\mathrm{dR}}{\mathrm{dt}}=r\left(T\right)R\left(1-\frac{R}{K\left(T\right)}\right)-f\left(R,T\right)∙C$$

where

$$f\left(R,T\right)=I_{\max}\left(T\right)∙\frac{R}{R+R_{0}}$$

This model has three equilibrium solutions, and we categorize them based on the presence or absence of the consumer and resource:

$$\text{Trivial}\;\left(\xi^{0,0}\right):R_{e},C_{e}=0$$

$$\text{Resource only}\;\left(\xi^{+,0}\right):R_{e}=K\left(T\right)\;\text{and}\;C_{e}=0$$

$$\text{Coexistence}\;\left(\xi^{+,+}\right),R_{e}=\frac{m\left(T\right)∙R_{0}}{\left(1-\delta \right)I_{\max}\left(T\right)-m\left(T\right)}\;\text{and}$$

$$\;C_{e}=\left(1-\frac{R_{e}}{K\left(T\right)}\right)\left(\frac{r\left(T\right)∙\left(1-\delta \right)}{m\left(T\right)}\right)R_{e}$$

Note that for coexistence, the consumer's equilibrium is written as a function of the resource equilibrium *R*_*e*_.

## References

[ele14086-bib-0001] Alexander, G.J. , Hanrahan, S.A. & Mitchell, D. (2012) Assimilation efficiency and gut passage time in an African elapid snake, Hemachatus haemachatus. African Journal of Herpetology, 61, 3–13.

[ele14086-bib-0002] Amarasekare, P. (2015) Effects of temperature on consumer–resource interactions. Journal of Animal Ecology, 84, 665–679. 25412342 10.1111/1365-2656.12320

[ele14086-bib-0003] Amarasekare, P. & Savage, V. (2012) A framework for elucidating the temperature dependence of fitness. The American Naturalist, 179, 178–191. 10.1086/663677 22218308

[ele14086-bib-0004] Angilletta, M. (2009) Thermal adaptation: A theoretical and empirical synthesis. New York: Oxford University Press.

[ele14086-bib-0077] Aranguren‐Gassis, M. , Kremer, C.T. , Klausmeier, C.A. & Litchman, E. (2019) Nitrogen limitation inhibits marine diatom adaptation to high temperatures. Ecology Letters, 22(11), 1860–1869. 10.1111/ele.13378 31429516

[ele14086-bib-0005] Auer, S.K. , Salin, A.M. , Rudolf, A. , Anderson, G.J. & Metcalfe, N.B. (2015) Flexibility in metabolic rate confers a growth advantage under changing food availability. Journal of Animal Ecology, 84(5), 1405–1411. 25939669 10.1111/1365-2656.12384 PMC4682473

[ele14086-bib-0006] Barbier, M. & Loreau, M. (2019) Pyramids and cascades: a synthesis of food chain functioning and stability. Ecology Letters, 22(2), 405–419. 30560550 10.1111/ele.13196 PMC6379062

[ele14086-bib-0007] Barneche, D.R. , Hulatt, C.J. , Dossena, M. , Padfield, D. , Woodward, G. , Trimmer, M. et al. (2021) Warming impairs trophic transfer efficiency in a long‐term field experiment. Nature, 592(7852), 76–79. 33647927 10.1038/s41586-021-03352-2

[ele14086-bib-0008] Bennett, A.F. & Lenski, R.E. (1993) Evolutionary adaptation to temperature II. Thermal niches of experimental lines of Escherichia coli. Evolution, 47(1), 1–12. 28568084 10.1111/j.1558-5646.1993.tb01194.x

[ele14086-bib-0009] Bernhardt, J.R. , Sunday, J.M. & O'Connor, M.I. (2018) Metabolic theory and the temperature‐size rule explain the temperature dependence of population carrying capacity. The American Naturalist, 192, 687–697. 10.1086/700114 30444656

[ele14086-bib-0010] Brett, J.R. (1971) Energetic responses of salmon to temperature. A study of some thermal relations in the physiology and freshwater ecology of sockeye salmon (*Oncorhynchus nerkd*). American Zoologist, 11, 99–113.

[ele14086-bib-0011] Brett, J.R. , Shelbourn, J.E. & Shoop, C.T. (1969) Growth rate and body composition of fingerling sockeye salmon, Oncorhynchus nerka, in relation to temperature and ration size. Journal of the Fisheries Board of Canada, 26(9), 2363–2394.

[ele14086-bib-0012] Brown, J.H. , Gillooly, J.F. , Allen, A.P. , Savage, V.M. & West, G.B. (2004) Toward a metabolic theory of ecology. Ecology, 85, 1771–1789.

[ele14086-bib-0013] Cohen, J.M. , Venesky, M.D. , Sauer, E.L. , Civitello, D.J. , McMahon, T.A. , Roznik, E.A. et al. (2017) The thermal mismatch hypothesis explains host susceptibility to an emerging infectious disease. Ecology Letters, 20, 184–193. 28111904 10.1111/ele.12720

[ele14086-bib-0014] Daugaard, U. , Petchey, O.L. & Pennekamp, F. (2019) Warming can destabilize predator–prey interactions by shifting the functional response from type III to type II. Journal of Animal Ecology, 88, 1575–1586. 31257583 10.1111/1365-2656.13053

[ele14086-bib-0015] Dell, A.I. , Pawar, S. & Savage, V.M. (2011) Systematic variation in the temperature dependence of physiological and ecological traits. Proceedings of the National Academy of Sciences, 108, 10591–10596. 10.1073/pnas.1015178108 PMC3127911 21606358

[ele14086-bib-0016] Dell, A.I. , Pawar, S. & Savage, V.M. (2014) Temperature dependence of trophic interactions are driven by asymmetry of species responses and foraging strategy. The Journal of Animal Ecology, 83, 70–84. 23692182 10.1111/1365-2656.12081

[ele14086-bib-0078] DeLong, J.P. & Vasseur, D.A. (2012) A dynamic explanation of size–density scaling in carnivores. Ecology, 93(3), 470–476. 10.1890/11-1138.1 22624202

[ele14086-bib-0017] Deutsch, C.A. , Tewksbury, J.J. , Huey, R.B. , Sheldon, K.S. , Ghalambor, C.K. , Haak, D.C. et al. (2008) Impacts of climate warming on terrestrial ectotherms across latitude. Proceedings of the National Academy of Sciences, 105, 6668–6672. 10.1073/pnas.0709472105 PMC2373333 18458348

[ele14086-bib-0018] Englund, G. , Ohlund, G. , Hein, C.L. & Diehl, S. (2011) Temperature dependence of the functional response. Ecology Letters, 14, 914–921. 21752171 10.1111/j.1461-0248.2011.01661.x

[ele14086-bib-0019] Fey, S.B. , Vasseur, D.A. , Alujević, K. , Kroeker, K.J. , Logan, M.L. , O'Connor, M.I. et al. (2019) Opportunities for behavioral rescue under rapid environmental change. Global Change Biology, 25, 3110–3120. 31148329 10.1111/gcb.14712

[ele14086-bib-0020] Finch, E.A. , T., Beale, M. , Chellappan, G. , Goergen, B.G. , Gadratagi, M.A.M. , Khan, A. , Rehman, I. , Rwomushana, A.K. , Sarma, K.A. , Wyckhuys, and D.J., Kriticos. 2021. The potential global distribution of the papaya mealybug, Paracoccus marginatus, a polyphagous pest. Pest Management Science, 77(3), pp.1361–1370. 33089608 10.1002/ps.6151 PMC7894313

[ele14086-bib-0021] Franken, O. , Huizinga, M. , Ellers, J. & Berg, M.P. (2018) Heated communities: large inter‐ and intraspecific variation in heat tolerance across trophic levels of a soil arthropod community. Oecologia, 186, 311–322. 29224117 10.1007/s00442-017-4032-z PMC5799326

[ele14086-bib-0022] Fronhofer, E.A. , D., Legrand, F. , Altermatt, A. , Ansart, S. , Blanchet, D. , Bonte, A. , Chaine, M. , Dahirel, F. , De Laender, J. , De Raedt, J. and Di Gesu, L. 2018. Bottom‐up and top‐down control of dispersal across major organismal groups. Nature Ecology & Evolution, 2(12), pp.1859–1863. 30397298 10.1038/s41559-018-0686-0

[ele14086-bib-0023] Fussmann, K.E. , Schwarzmüller, F. , Brose, U. , Jousset, A. & Rall, B.C. (2014) Ecological stability in response to warming. Nature Climate Change, 4, 206–210.

[ele14086-bib-0024] Gillooly, J.F. , McCoy, M.W. & Allen, A.P. (2007) Effects of metabolic rate on protein evolution. Biology Letters, 3(6), 655–660. 17911050 10.1098/rsbl.2007.0403 PMC2391229

[ele14086-bib-0025] Gilman, S.E. , Urban, M.C. , Tewksbury, J. , Gilchrist, G.W. & Holt, R.D. (2010) A framework for community interactions under climate change. Trends in Ecology & Evolution, 25(6), 325–331. 20392517 10.1016/j.tree.2010.03.002

[ele14086-bib-0026] Ginzburg, L.R. & Akcakaya, H.R. (1992) Consequences of ratio‐dependent predation for steady‐state properties of ecosystems. Ecology, 73(5), 1536–1543.

[ele14086-bib-0027] Gjelland, K.Ø. , Bøhn, T. & Amundsen, P.‐A. (2007) Is coexistence mediated by microhabitat segregation? An in‐depth exploration of a fish invasion. Journal of Fish Biology, 71, 196–209.

[ele14086-bib-0028] Hairston, N.G. , Smith, F.E. & Slobodkin, L.B. (1960) Community structure, population control, and competition. The American Naturalist, 94(879), 421–425.

[ele14086-bib-0029] Holt, R.D. (2009) Bringing the Hutchinsonian niche into the 21st century: ecological and evolutionary perspectives. Proceedings of the National Academy of Sciences, 106, 19659–19665. 10.1073/pnas.0905137106 PMC2780934 19903876

[ele14086-bib-0030] Huey, R.B. & Kingsolver, J.G. (2019) Climate warming, resource availability, and the metabolic meltdown of ectotherms. The American Naturalist, 194, E140–E150. 10.1086/705679 31738103

[ele14086-bib-0031] Huey, R.B. & Slatkin, M. (1976) Cost and benefits of lizard thermoregulation. The Quarterly Review of Biology, 51(3), 363–384. 981504 10.1086/409470

[ele14086-bib-0032] Khelifa, R. , Blanckenhorn, W.U. , Roy, J. , Rohner, P.T. & Mahdjoub, H. (2019) Usefulness and limitations of thermal performance curves in predicting ectotherm development under climatic variability. Journal of Animal Ecology, 88, 1901–1912. 31365760 10.1111/1365-2656.13077

[ele14086-bib-0033] Kingsolver, J.G. (2009) The well‐temperatured biologist. The American Naturalist, 174, 755–768. 10.1086/648310 19857158

[ele14086-bib-0034] Kordas, R.L. , Harley, C.D. & O'Connor, M.I. (2011) Community ecology in a warming world: the influence of temperature on interspecific interactions in marine systems. Journal of Experimental Marine Biology and Ecology, 400(1–2), 218–226.

[ele14086-bib-0035] Kremer, C.T. , Fey, S.B. , Arellano, A.A. & Vasseur, D.A. (2018) Gradual plasticity alters population dynamics in variable environments: thermal acclimation in the green alga Chlamydomonas reinhartdii. Proceedings Biological Sciences, 285, 20171942. 29321297 10.1098/rspb.2017.1942 PMC5784192

[ele14086-bib-0036] Lancaster, L.T. & Humphreys, A.M. (2020) Global variation in the thermal tolerances of plants. Proceedings of the National Academy of Sciences, 117(24), 13580–13587. 10.1073/pnas.1918162117 PMC7306813 32482870

[ele14086-bib-0037] Lemoine, N.P. (2019) Considering the effects of temperature × nutrient interactions on the thermal response curve of carrying capacity. Ecology, 100, e02599. 30620393 10.1002/ecy.2599

[ele14086-bib-0038] Lister, B.C. & Garcia, A. (2018) Climate‐driven declines in arthropod abundance restructure a rainforest food web. Proceedings of the National Academy of Sciences, 115(44), E10397–E10406. 10.1073/pnas.1722477115 PMC6217376 30322922

[ele14086-bib-0039] Lotka, A.J. (1925) Elements of physical biology. Baltimore: Williams & Wilkins.

[ele14086-bib-0040] Mallet, J. (2012) The struggle for existence. How the notion of carrying capacity, K, obscures the links between demography, Darwinian evolution and speciation. Evolutionary Ecology Research, 14, 627–665.

[ele14086-bib-0041] Nowakowski, A.J. , Whitfield, S.M. , Eskew, E.A. , Thompson, M.E. , Rose, J.P. , Caraballo, B.L. et al. (2016) Infection risk decreases with increasing mismatch in host and pathogen environmental tolerances. Ecology Letters, 19, 1051–1061. 27339786 10.1111/ele.12641

[ele14086-bib-0042] O'Connor, M.I. (2009) Warming strengthens an herbivore–plant interaction. Ecology, 90, 388–398. 19323223 10.1890/08-0034.1

[ele14086-bib-0043] Pan, T. , Zou, X. , Liu, Y. , Wu, S. & He, G. (2017) Contributions of climatic and non‐climatic drivers to grassland variations on the Tibetan plateau. Ecological Engineering, 108, 307–317.

[ele14086-bib-0044] Pawar, S. , Dell, A.I. & Savage, V.M. (2012) Dimensionality of consumer search space drives trophic interaction strengths. Nature, 486, 485–489. 22722834 10.1038/nature11131

[ele14086-bib-0045] Pearson, R.G. & Dawson, T.P. (2003) Predicting the impacts of climate change on the distribution of species: are bioclimate envelope models useful? Global Ecology and Biogeography, 12, 361–371.

[ele14086-bib-0046] Petchey, O.L. , McPhearson, P.T. , Casey, T.M. & Morin, P.J. (1999) Environmental warming alters food‐web structure and ecosystem function. Nature, 402, 69–72.

[ele14086-bib-0047] Pinsky, M.L. , Eikeset, A.M. , McCauley, D.J. , Payne, J.L. & Sunday, J.M. (2019) Greater vulnerability to warming of marine versus terrestrial ectotherms. Nature, 569, 108–111. 31019302 10.1038/s41586-019-1132-4

[ele14086-bib-0048] Porter, W.P. , Mitchell, J.W. , Beckman, W.A. & DeWitt, C.B. (1973) Behavioral implications of mechanistic ecology: thermal and behavioral modeling of desert ectotherms and their microenvironment. Oecologia, 13, 1–54. 28307981 10.1007/BF00379617

[ele14086-bib-0049] Rall, B.C. , Vucic‐Pestic, O. , Ehnes, R.B. , Emmerson, M. & Brose, U. (2010) Temperature, predator–prey interaction strength and population stability. Global Change Biology, 16, 2145–2157.

[ele14086-bib-0050] Rosenzweig, M.L. & MacArthur, R.H. (1963) Graphical representation and stability conditions of predator–prey interactions. The American Naturalist, 97, 209–223.

[ele14086-bib-0051] Savage, V.M. , Gillooly, J.F. , Brown, J.H. , West, G.B. & Charnov, E.L. (2004) Effects of body size and temperature on population growth. The American Naturalist, 163, 429–441. 10.1086/381872 15026978

[ele14086-bib-0052] Schulte, P.M. , Healy, T.M. & Fangue, N.A. (2011) Thermal performance curves, phenotypic plasticity, and the time scales of temperature exposure. Integrative and Comparative Biology, 51, 691–702. 21841184 10.1093/icb/icr097

[ele14086-bib-0053] Seebacher, F. , White, C.R. & Franklin, C.E. (2015) Physiological plasticity increases resilience of ectothermic animals to climate change. Nature Climate Change, 5(1), 61–66.

[ele14086-bib-0054] Sentis, A. , Hemptinne, J.‐L. & Brodeur, J. (2013) Effects of simulated heat waves on an experimental plant–herbivore–predator food chain. Global Change Biology, 19, 833–842. 23504840 10.1111/gcb.12094

[ele14086-bib-0055] Sentis, A. , Morisson, J. & Boukal, D.S. (2015) Thermal acclimation modulates the impacts of temperature and enrichment on trophic interaction strengths and population dynamics. Global Change Biology, 21(9), 3290–3298. 25808556 10.1111/gcb.12931

[ele14086-bib-0056] Sherry, T.W. (2021) Sensitivity of tropical insectivorous birds to the Anthropocene: a review of multiple mechanisms and conservation implications. Frontiers in Ecology and Evolution, 9.

[ele14086-bib-0057] Sinclair, B.J. , Marshall, K.E. , Sewell, M.A. , Levesque, D.L. , Willett, C.S. , Slotsbo, S. et al. (2016) Can we predict ectotherm responses to climate change using thermal performance curves and body temperatures? Ecology Letters, 19, 1372–1385. 27667778 10.1111/ele.12686

[ele14086-bib-0058] Smith, D.J. & Amarasekare, P. (2018) Toward a mechanistic understanding of thermal niche partitioning. The American Naturalist, 191, E57–E75.

[ele14086-bib-0059] Sohlström, E.H. , Archer, L.C. , Gallo, B. , Jochum, M. , Kordas, R.L. , Rall, B.C. et al. (2021) Thermal acclimation increases the stability of a predator–prey interaction in warmer environments. Global Change Biology, 27(16), 3765–3778. 34009702 10.1111/gcb.15715

[ele14086-bib-0060] Strong, D.R. (1992) Are trophic cascades all wet? Differentiation and donor‐control in speciose ecosystems. Ecology, 73, 747–754.

[ele14086-bib-0061] Sunday, J.M. , Bates, A.E. , Kearney, M.R. , Colwell, R.K. , Dulvy, N.K. , Longino, J.T. et al. (2014) Thermal‐safety margins and the necessity of thermoregulatory behavior across latitude and elevation. Proceedings of the National Academy of Sciences, 111, 5610–5615. 10.1073/pnas.1316145111 PMC3992687 24616528

[ele14086-bib-0062] Synodinos, A.D. , Haegeman, B. , Sentis, A. & Montoya, J.M. (2021) Theory of temperature‐dependent consumer–resource interactions. Ecology Letters, 24(8), 1539–1555. 34120390 10.1111/ele.13780 PMC7614043

[ele14086-bib-0063] Theus, M.E. , Layden, T.J. , McWilliams, N. , Crafton‐Tempel, S. , Kremer, C.T. & Fey, S.B. (2022) Photoperiod influences the shape and scaling of freshwater phytoplankton responses to light and temperature. Oikos, 2022, e08839.

[ele14086-bib-0064] Thomas, M.K. , Aranguren‐Gassis, M. , Kremer, C.T. , Gould, M.R. , Anderson, K. , Klausmeier, C.A. et al. (2017) Temperature–nutrient interactions exacerbate sensitivity to warming in phytoplankton. Global Change Biology, 23(8), 3269–3280. 28132424 10.1111/gcb.13641

[ele14086-bib-0065] Tilman, D. (1977) Resource competition between plankton algae: an experimental and theoretical approach. Ecology, 58, 338–348.

[ele14086-bib-0066] Urban, M.C. , Bocedi, G. , Hendry, A.P. , Mihoub, J.‐B. , Peer, G. , Singer, A. et al. (2016) Improving the forecast for biodiversity under climate change. Science, 353, 1113. 10.1126/science.aad8466 27609898

[ele14086-bib-0067] Uszko, W. , Diehl, S. , Englund, G. & Amarasekare, P. (2017) Effects of warming on predator–prey interactions–a resource‐based approach and a theoretical synthesis. Ecology Letters, 20, 513–523. 28266168 10.1111/ele.12755

[ele14086-bib-0068] Vasseur, D.A. (2020) Climate change: studying the effects of temperature on population and community dynamics. Theoretical Ecology, Concepts and Applications. New York: Oxford University Press.

[ele14086-bib-0069] Vasseur, D.A. , DeLong, J.P. , Gilbert, B. , Greig, H.S. , Harley, C.D.G. , McCann, K.S. et al. (2014) Increased temperature variation poses a greater risk to species than climate warming. Proceedings of the Royal Society B: Biological Sciences, 281, 20132612. 10.1098/rspb.2013.2612 PMC3924069 24478296

[ele14086-bib-0070] Vasseur, D.A. & McCann, K.S. (2005) A mechanistic approach for modeling temperature‐dependent consumer‐resource dynamics. The American Naturalist, 166, 184–198. 10.1086/431285 16032573

[ele14086-bib-0071] Voigt, W. , Perner, J. , Davis, A.J. , Eggers, T. , Schumacher, J. , Bährmann, R. et al. (2003) Trophic levels are differentially sensitive to climate. Ecology, 84, 2444–2453.

[ele14086-bib-0072] Volterra, V. (1926) Fluctuations in the abundance of a species considered mathematically. Nature, 118(2972), 558–560.

[ele14086-bib-0073] Woods, H.A. , Kingsolver, J.G. , Fey, S.B. & Vasseur, D.A. (2018) Uncertainty in geographical estimates of performance and fitness. Methods in Ecology and Evolution, 9, 1996–2008.

[ele14086-bib-0074] Xu, X.‐F. & Ji, X. (2006) Ontogenetic shifts in thermal tolerance, selected body temperature and thermal dependence of food assimilation and locomotor performance in a lacertid lizard, Eremias brenchleyi. Comparative biochemistry and physiology. Part A, Molecular & Integrative Physiology, 143, 118–124. 10.1016/j.cbpa.2005.11.004 16380280

[ele14086-bib-0075] Yodzis, P. & Innes, S. (1992) Body size and consumer‐resource dynamics. The American Naturalist, 139, 1151–1175.

[ele14086-bib-0076] Zalasiewicz, J. , Waters, C.N. , Summerhayes, C.P. , Wolfe, A.P. , Barnosky, A.D. , Cearreta, A. et al. (2017) The working group on the anthropocene: summary of evidence and interim recommendations. Anthropocene, 19, 55–60.

